# Robustness vs. flexibility: how do external inputs shape the activity in a data-based layered cortical network model?

**DOI:** 10.1186/1471-2202-12-S1-P74

**Published:** 2011-07-18

**Authors:** Tobias C Potjans, Markus Diesmann

**Affiliations:** 1 Institute of Neuroscience and Medicine (INM-6), Research Center Juelich, Germany; 2 RIKEN Computational Science Research Program, Wako-shi, Saitama, Japan; 3 RIKEN Brain Science Institute, Wako-shi, Saitama, Japan

## 

We developed a model of the layered cortical network that integrates a major body of the available physiological and anatomical connectivity data on the cortical microcircuit [1]. The model extents the well-known balanced random network model [2,3] which reproduces the asynchronous irregular firing regime observed in vivo. The data-based multi-layered extension, in addition, yields cell-type specific firing rates and activation patterns after transient stimulation in accordance with in vivo recordings in awake animals, see e.g. [4].

In mono-layered balanced random network models the external input can be characterized by a single parameter, the external background firing rate. In contrast, the multi-layered network requires the specification of background inputs for every layer. We therefore distinguish the number of inputs a neuron of a given cell type receives and the background firing rate per external synapse. The experimentally observed cell-type specific firing rates are more accurately reproduced by a data-based estimate of the number of external inputs than by a layer-independent input parameterization. Still, it remains unclear how the overall contributions of background activity to the different layers shape the activity of the network.

Here, we analyze the dependence of the activity in the full-scale local cortical network model on the external inputs. We apply large-scale parameter scans in NEST [5] to study the effect of changes in the overall excitation of the network as well as of layer-specific modifications. Firstly, we find that, confronted with increasing overall excitation, the excitatory populations in the different layers react partly contrary, with rate increases or decreases. Secondly, we test for the robustness of the cell-type specific distribution of spontaneous firing rates: we consider randomly selected numbers of external inputs, constraint by data-based estimates, and reveal that the experimental activity measurements –low firing rates in layer 2/3 and 6, intermediate rates in layer 4 and highest activity in layer 5– are reproduced in around 80% of the trials. We test whether this remarkable robustness comes at the price of a reduced flexibility of the network, but find that in the same parameter setting the activity of principal cells strongly depends on the balance of the excitation in a given layer, reflecting a selective activity gain. Furthermore, we investigate the transmission properties to transient inputs and their dependence on the network state.

## Conclusion

The layered cortical network robustly reproduces the activity patterns observed in vivo for a wide range of external input configurations: the cortical microcircuit dynamically reconfigures its activity and preserves a default state. This robustness does not affect the ability of the network to process transients and changes in the balance of external inputs. This microcircuit mechanism may provide an explanation for the similarity of the spontaneous firing rates that have been observed, especially in layer 2/3, in various species and areas [4].

Supported by the Helmholtz Alliance on Systems Biology, JUGENE Grant JINB33, the Next-Generation Supercomputer Project of MEXT, Japan, and EU Grant 269921 (BrainScaleS).
